# Commentary: “Personality and Intentional Binding: An Exploratory Study Using the Narcissistic Personality Inventory”

**DOI:** 10.3389/fnhum.2015.00325

**Published:** 2015-06-03

**Authors:** Giancarlo Dimaggio, Paul H. Lysaker

**Affiliations:** ^1^Centro di Terapia Metacognitiva Interpersonale, Rome, Italy; ^2^Richard L. Roudebush VA Medical Center (116H), Indianapolis, IN, USA; ^3^Indiana University School of Medicine, Indianapolis, IN, USA

**Keywords:** narcissism, agency, narcissistic personality disorder, vulnerable narcissism, personality disorders

Hascalovitz and Obhi (2015) examined whether narcissism was related to a disturbed sense of agency. Using the construct of intentional binding, which is thought to be a core element of agency, they sought to determine whether the speed with which a voluntary action was perceived to have a consequent effect differed according to levels of narcissism. They hypothesized that higher levels of narcissism will predict hyper-agency or increased sense that one’s actions will have immediate effects on the world. Hascalovitz and Obhi note that this is consistent with observations that patients with narcissistic personality disorder (NPD) often report that they feel in control of their environment and tend to err on the side of an omnipotence, which they believe should result in admiration from others. Indeed, persons with NPD are often described as feeling entitled, and quick to take impulsive action if they are not perceived as they think they should, leading frequently to ruptures in relationships that previously appeared solid.

Of note, there are many long-standing hypotheses about the potential relationship between narcissism and agency, which suggest a different view. Kohut (1971), for instance, observed that a core feature of NPD is a hidden sense of fundamental powerlessness, which involves being relatively unable to influence others within the flow of life. Others have suggested that a lack of agency in NPD leads to a loss of touch with the person’s deepest wishes resulting in the experience of being flat, devitalized, and empty (Modell, 1984; Dimaggio et al., 2002; Dimaggio and Attinà, 2012). This is consistent with clinical reports that recovery from NPD involves increased agency in domains other than social rank, such as creativity and playfulness (Dimaggio et al., 2015).

How then can these results be reconciled with the larger literature? Even with the limitations that Hascalovitz and Obhi (2015) acknowledge prevent the generalizability of their study, their findings suggest that with narcissism comes a heightened sense of effectiveness and control over willful action. One possibility suggested by clinical reports (Ronningstam, 2009; Links, 2015) is that persons with NPD alternate from hyper-agency to passivity and flatness. We would like to explore a different possibility, specifically one that distinguishes instrumental agency, or agency in dealing with the non-personal world, from interpersonal agency or agency, which involves affecting how others think and behave. In other words, perhaps these results help us to see that in narcissism there is not merely an excess or deficit in agency but a paradox in which there are high levels of instrumental agency complimented by low levels of interpersonal agency.

Persons with narcissism may have an initially high appraisal of their abilities to complete tasks effectively (Kohut, 1971), but then they may doubt that others will share this view. Therefore, despite a kind of heightened intentional binding, they should expect others will not properly admire their effectiveness and control, leading back to reduction in their sense of agency generated by over-reliance on the judgment of others and ending up in passivity. Indeed, we can well imagine how then the narcissist feels so painfully and deeply powerless. They alternate from perceiving that they possess considerable abilities to affect the world in instrumental ways, to the perception that others do not share that view and then believe that the negative perceptions of other people cannot be affected. Thus, in narcissism, we hypothesize that there may come a continuous oscillation between high instrumental agency and low interpersonal agency. This would be consistent with how relationships can be frustrating for patients with NPD and also how relationships can be so quickly and irreparably ruptured. Alternative ideas cannot be ruled out. For one, given the chance that two subtypes of narcissism likely exist (Zeigler-Hill et al., 2008), it is possible that the vulnerable subtype is more closely linked to reduced agency, for example when facing a romantic rejection which affects an already unstable self-esteem (Zeigler-Hill et al., 2011), while the grandiose type could experience hyper-agency.

The possibility of a continuous oscillation between high instrumental agency and low interpersonal agency may point to questions for future research. For one, it would seem possible to measure both self-appraisal of personal effectiveness in instrumental laboratory tasks and also the extent to which participants think others will agree with their appraisal of their abilities. It would also seem important to use tasks that involve appraisals of real-life social outcomes. In addition, there may be an even more nuanced set of relationships at play. As observed in session, persons with narcissism can experience rapid shifts in their states of mind (Dimaggio et al., 2008) as determined by interpersonal events. It has been suggested (Kohut, 1971; Ronningstam, 2009; Dimaggio et al., 2014; Pincus et al., 2014) that swings between an inflated sense of personal worth and a sense of virtual non-existence and failure largely depend on the presence of support and admiration from the environment. Thus, a lack of interpersonal agency may result in changes in self-appraisal of instrumental agency. This suggests that laboratory tasks seeking to measure agency in narcissism requires appropriate priming. One hypothesis is that after having been primed with experiences of social or romantic rejection, narcissistic-related oscillations in the sense of agency for instrumental tasks would appear. After social rejection, for example some high in grandiose narcissism may experiment increased sense of agency, while those with vulnerable narcissism may resemble the profile of persons with depression or low self-esteem and experience diminished agency.

Finally, a range of other questions also exist including whether what hyper-agency observed here is a function of just having a personality disorder in general or specific to this one kind of phenomenon. Work is thus needed, for example, with patients with dependent or avoidant traits (Dimaggio et al., 2015). As there may be different forms of narcissism, work is needed to determine whether agency is affected in similar ways in different manifestations of the condition or even more closely linked to some aspects of narcissism than others.

## Conflict of Interest Statement

The authors declare that the research was conducted in the absence of any commercial or financial relationships that could be construed as a potential conflict of interest.
